# The Effect of Vitamin D3 Intervention on the Association Among Vitamin D3, Adiponectin, and Body Mass Index in Pregnant Women With Gestational Diabetes

**DOI:** 10.7759/cureus.43506

**Published:** 2023-08-15

**Authors:** Amna Nadeem, Muniza Saeed, Ayesha Sadiqa, Hira Moin, Qudsia U Khan

**Affiliations:** 1 Physiology, CMH Lahore Medical College and Institute of Dentistry, Lahore, PAK; 2 Medicine and Surgery, National University of Medical Sciences (NUMS), Rawalpindi, PAK; 3 Physiology, Ameer-ud-Din Medical College, Lahore, PAK; 4 Physiology, National University of Sciences and Technology (NUST), Islamabad, PAK; 5 Physiology, CMH Lahore Medical and Dental College, Lahore, PAK

**Keywords:** body mass index, pregnant women, adiponectin, vitamin d supplementation, gestational diabetes

## Abstract

Introduction: Vitamin D3 (VD3) deficiency is a strong predictor of gestational diabetes. Therefore, VD3 supplementation during the antenatal period could prevent the development of gestational diabetes via its effects on insulin secretion, insulin sensitivity, body mass index (BMI), and adiponectin production.

Objectives: To observe the effect of VD3 supplementation on adiponectin and BMI and to explore the effect of VD3 supplementation on the association among VD3, adiponectin, and BMI in pregnant women with gestational diabetes.

Methods: A randomized control trial was performed after receiving consent at Postgraduate Medical Institute, Lahore. Subjects at 20-26 weeks of gestation with gestational diabetes and with a deficiency/insufficiency of VD3 were included. The study excluded those who were smokers, had multiple pregnancies, or had other gestational complications. Subjects were categorized into interventional (VD3 supplementation) and control groups. The institutional ethical committee approved the study. Serum samples were used for enzyme-linked immunosorbent assay estimation of VD3 and adiponectin levels. Statistical Product and Service Solutions (SPSS) (IBM SPSS Statistics for Windows, Version 21.0, Armonk, NY) software was used to analyze data. Student t-tests were applied to compare quantitative variables, and Chi-square tests were utilized to compare qualitative variables. Pearson’s correlation and linear regressions were performed to explore the association. At a 95% confidence interval, a p-value of ≤0.05 was taken as significant.

Results: With an increase in serum VD3 levels, a decrease in serum adiponectin level was observed in pregnant women with gestational diabetes (interventional group: r = -0.088, p = 0.74); however, after the intervention of VD3 supplementation in the same subjects, an increase in serum adiponectin level was noted with an increase in VD3 (interventional group: r = 0.273, p = 0.28). A significant direct relationship was found between BMI and adiponectin in the same study population (interventional group: r = 0.7, p = 0.001). Interestingly, after the intervention, BMI tends to be less likely to increase adiponectin levels (interventional group: r = 0.09, p = 0.73). Moreover, an inverse association was exhibited between BMI and VD3 levels in all the study groups before intervention (control group: r = -0.07, p = 0.78; interventional group: r = -0.035, p = 0.89) and after intervention (interventional group: r = -0.12, p = 0.65), except in the control group after the intervention span, where BMI mildly raises the VD3 levels (r = -0.12, p = 0.65).

Conclusion: BMI increases with an increase in serum adiponectin levels in gestational diabetic women, but after VD3 supplementation, BMI was less likely to influence adiponectin. Also, with an increase in BMI, a decrease in the VD3 in all study groups was observed except in the control group after VD3 supplementation.

## Introduction

Gestational diabetes mellitus (GDM) is a metabolic disorder with an estimated 1-28% prevalence in the global pregnant female population [1]. GDM can be defined as elevated glucose levels detected for the first time during pregnancy [2]. This is the most widespread obstetric pathology and has the highest prevalence in overweight and obese females [3]. The maternal complications of GDM include increased risk of pre-eclampsia, cesarean section, and operative vaginal deliveries. Neonates can also be affected by adverse complications such as macrosomia, hypoglycemia, and hyperbilirubinemia [3]. Moreover, GDM increases the likelihood of diabetes mellitus and glucose intolerance, cardiovascular diseases, and obesity later in the lives of mother and child [2,4]. The prevalence of GDM has increased worldwide in recent years; a study conducted in 2022 reported a prevalence of 9.47% in Pakistan [4]. The pathophysiology of GDM includes each participant was gone through medical history, general physical exam, and routine antenatal examination [5]. Furthermore, the total mass of adipose tissue, a chief endocrine organ, increases during pregnancy. This hypertrophy of adipose tissue leads to the dysregulated synthesis of adipokines, particularly leptin, and adiponectin, which chiefly contribute to the pathogenesis of GDM [6]. A study by Deca et al. suggests that adiponectin production rises with a decrease in body mass and declines in obese individuals, thus increasing the risk of atherogenesis and metabolic syndrome [7]. Likewise, during normal pregnancy, adiponectin levels are progressively reduced. However, in GDM, there is an excessive decline in its levels [8]. Additionally, the production of pro-inflammatory cytokines such as TNF-a and IL-6 is augmented in GDM, which further impedes adiponectin synthesis [9]. Adiponectin enhances insulin sensitivity by increasing glucose utilization and decreasing hepatic glucose production [10]. Consequently, a reduction in the concentration of adiponectin (particularly high-molecular-weight adiponectin) can predict and contribute to the pathogenesis of GDM [11].

Vitamin D3 (VD3) is a well-known steroid compound that helps maintain calcium balance and promote bone mineralization. Pregnant women with deficient VD3 have an increased risk of developing pregnancy-related disorders such as GDM, pre-eclampsia, and pre-term labor [12]. Numerous extra-skeletal functions of VD3 have also been identified, including modulation of the immune system and glucose metabolism, prevention of cardiovascular diseases, and reduction of mortality [12,13]. With such considerable effects on several body functions, VD3 deficiency has become a health burden that affects a wide range of pregnant female populations worldwide. The deficiency of this vitamin is linked to an increased incidence of diabetes mellitus type 2 and obesity in females of advanced reproductive age [13]. VD receptors have been identified in almost every type of cell in the body, suggesting the potential role of VD3 in regulating various metabolic pathways. VD3 regulates the function of β-pancreatic cells and insulin sensitivity in different body tissues, and there is a 1.29-fold increase in the risk of GDM with every 5 ng/mL decrease in VD3 levels [14]. Hosseinzadeh et al. reported that the levels of VD3 are negatively associated with body mass index (BMI). The mechanisms by which VD3 can alleviate adiposity and BMI in obese individuals include the activation of calcium-dependent apoptosis in adipose tissue and reducing the differentiation of pre-adipocytes to adipocytes [15].

It has also been observed that VD3 synthesis takes place in placental tissue and female reproductive structures such as the ovaries and endometrium and plays a significant role in the maintenance of pregnancy as it affects glucose homeostasis and placental and immunological cell functioning [16]. Moreover, VD3 supplementation modulates the expression of adipokines in visceral fat, and there is a positive correlation between serum adiponectin and serum VD3 levels [17]. Here, the current study aims to observe the effect of VD3 supplementation on adiponectin and BMI levels and explore the effect of VD3 supplementation on the association among VD3, adiponectin, and BMI in pregnant women with gestational diabetes.

## Materials and methods

After obtaining their written consent, a randomized controlled trial was carried out with 34 participants. All the study participants were pregnant women and were diagnosed with GDM as well as VD3 deficiency. The study was approved by the institutional ethical committee of the Post Graduate Medical Institute, Lahore. All the study participants were patients of the Gynaecology and Obstetrics Department of Lahore General Hospital Lahore and Lady Aitchison Hospital Lahore.

The sample size was calculated through Cohen’s D formula. A non-probable purposive sampling technique was used after randomization by balloting to select the study population. All the study participants were randomly stratified into two categories, the interventional and the control group; each group had 17 participants. An intramuscular dose of 200,000 IU of VD3 was injected into the interventional group, whereas the control group was not provided with any VD3 intervention. Serum samples were collected from both groups before intervention. Then, second-time serum samples were taken from the interventional group after a waiting period of three months post-injection of VD3. In the control group, as no intervention was performed, serum samples were taken again after three months. All the participants of both study groups were taking routine antenatal oral vitamin supplements.

After institutional approval, the study was completed in one year, from June 2020 to June 2021. The participants were 21-32 years old, with an average (± SD) of 26.12 ± 2.64. All the subjects were of gestational age of 20-26 weeks, diagnosed with GDM confirmed through values of an oral glucose tolerance test, i.e. those who scored ≥100 mg per dL and who also had a deficiency or insufficiency of VD3 (<29 ng per mL) were included in the study. Those who were smokers had multiple pregnancies, had other gestational complications, had related fetal anomalies, or were already diagnosed with diseases such as diabetes mellitus type 2 or hypertension were excluded from the study.

GDM was confirmed via OGTT (oral glucose tolerance test ≥100 mg/dL). Serum vitamin D <29 ng/mL was considered vitamin D-deficient. A medical history was taken from each study participant, and a general physical examination, including height and weight measurement, was performed. About 5 mL of blood sample was taken from each participant utilizing the aseptic technique. Then sera were collected through centrifugation of 15 minutes at a speed of 1,500 × g to monitor serum adiponectin levels and VD3 concentrations. All the serum samples were stored at -80° C before their quantitative estimation via enzyme-linked immunosorbent assay technique to assess serum VD3 and adiponectin levels.

The data were analyzed using Statistical Product and Service Solutions (SPSS) (IBM SPSS Statistics for Windows, Version 21.0, Armonk, NY) software. For the comparative analysis of two quantitative variables, the student t-test was applied, and for the qualitative variable comparison, the Chi-square test was used. Pearson’s correlation and linear regression were performed to find any association between the two study variables. At a 95% confidence interval, a p-value of ≤0.05 was taken as statistically significant.

## Results

A statistically insignificant difference (p = 0.054) was noted between the control and the interventional group’s average age (± SD). The mean (± SD) BMI in kg/m² of the total study population before intervention was collectively 23.21 ± 1.17, with 21.0 as the least and 25.3 being the highest value of BMI. Again, an insignificant difference with p = 0.726 was detected when the average mean (± SD) of both study groups, i.e. the control and the interventional groups, was compared. However, when the BMI was calculated again after the VD3 intervention and a waiting period of three months, a very significant difference (p = 0.009) was observed between the two groups. Moreover, a 5.32% decreased BMI was found in the interventional group compared to the control group (Table 1).

**Table 1:** Demographic characteristics of the study population *BMI = body mass index *p-value of ≤0.05 was taken as statistically significant *1 USD = 150 PKR

Demographic variables	Total subjects (n = 34)	Control (n = 17)	Interventional (n = 17)	p-value
Age (year) Mean ± SD	26.12 ± 2.64	27.0 ± 1.7	25.24 ±3.2	0.054
BMI* (kg/m²) Mean ± SD	23.21 ± 1.17	23.23 ±1.28	23.38 ± 1.14	0.726
BMI after 3 months (kg/m²) Mean ± SD	28.52 ± 1.76	29.30 ± 1.87	27.74 ± 1.33	0.009*
Skin tone n (%)	Very light	2 (11.8)	6 (35.3)	0.157
	Intermediate	4 (23.5)	5 (29.4)	
	Mediterranean	11 (64.7)	6 (35.3)	
Income (PKR* per month) n (%)	5000-10,000 PKR (33-67 USD)	6 (35.3)	5 (29.4)	0.931
	10,000-15,000 PKR (67-100 USD)	9 (52.9)	10 (58.8)	
	15,000-25,000* (100-167 USD)	2 (11.8)	2 (11.8)	
Education n (%)	Up to matriculation	12 (70.6)	8 (47.1)	0.296
	Intermediate to graduation	5 (29.4)	9 (52.9)	
Use of abaya n (%)	Yes	11 (64.7)	11 (64.7)	1.000
	No	6 (35.3)	6 (35.3)	
Sun exposure n (%)	Yes	6 (35.3)	7 (41.2)	1.000
	No	11 (64.7)	10 (58.8)	
Parity n (%)	Nulliparous	8 (47.1)	12 (70.6)	0.235
	Para 1	6 (35.3)	3 (17.6)	
	Para 2	3 (17.6)	1 (5.9)	
	Para 3	0	1 (5.9)	

The study participants were classified into three categories of skin tone, i.e. very light, intermediate, and Mediterranean skin tones. Noticeably, the control group’s highest number (11 out of 17) had Mediterranean skin tones, and the least (only two out of 17) had very light skin tones. Of the 17 interventional participants, six had very light skin tones, six had Mediterranean skin tones, and the least number (five) of the interventional participants had an intermediate skin tone. No significant difference (χ2 = 3.70, p = 0.157) was expressed between the control and the interventional groups regarding their skin tones (Table 1).

The study population was classified into three strata regarding their monthly income (ranging from 5,000 to 25,000 PKR per month or from 33 to 167 USD per month). The maximum number of participants fell in the income class of 10,000-15,000 PKR per month or 67-100 USD per month, in both the control (nine of 17) and the interventional group (10 out of 17). The fewest participants belonged to the income class of 15,000-25,000 PKR per month or 100-167 USD per month, in both the study groups, i.e. two of 17 from each group. The statistical difference between both comparative groups was found insignificant (χ2 = 0.144, p = 0.931) concerning their income status (Table 1).

When the study subjects were analyzed for their history of gravidity, it was seen that most subjects were nulliparous; the control group was eight out of 17, and the interventional group was 12 out of 17. None from the control group belonged to para 3, and only one out of 17 belonged to para 3 from the interventional group. The statistical difference for parity between the control and the interventional groups was also insignificant (χ2 = 4.26, p = 0.235) (Table 1).

Looking at education, the study population was divided into two levels from up to matriculation until graduation. Most participants from the control group (12 out of 17) were educated up to matriculation, while a larger number of participants from the interventional group (nine out of 17) were between the higher secondary and graduation levels of education. In this regard, no significant difference was revealed between the control and the interventional study groups (χ2 = 1.09, p = 0.296) (Table 1).

When all the study subjects were queried about sunlight exposure, the majority from each comparative group (11 from the control group and 10 from the interventional group) reported in the negative, as most were homemakers and remained inside most of the time. No statistical difference was found between the two study groups concerning sunlight exposure (χ2 = 0.0, p = 1.000). Similarly, when they were asked about wearing an ‘abaya (clothing covering the entirety of the body apart from the face)’, most of the subjects in both comparative groups used to wear one, i.e. 11 in each group wore an abaya. Again, no significant difference was found in using an abaya between the control and the interventional group (χ2 = 0.0, p = 1.000) (Table 1).

The serum levels of VD3 (ng/mL) in the controls showed only a 6.05% raised value after a period of three months, and the statistical difference between the two compared values (before and after the three-month period) was proved insignificant (p = 0.44). When a similar comparison was observed before and after the intervention (VD3 injection followed by a waiting period of three months) in the interventional group, a remarkable increase of 120% was expressed in the value of VD3 after the intervention. The difference between before and after values of serum VD3 was very significant (p = 0.000) in the interventional group (Table 2).

**Table 2:** Statistical comparison of biochemical parameters before and after three months of intervention in study groups *BMI = body mass index *p-value of ≤0.05 was taken as statistically significant

Study parameters	Study groups	Before intervention		After 3 months of intervention		p-value
		n	Mean ± SD	n	Mean ± SD	
Vitamin D3 (ng/mL)	Control	17	16.03 ± 4.3	17	17.0 ± 4.8	0.44
	Interventional	17	15.4 ± 3.8	17	33.9 ± 6.2	0.000*
Adiponectin (μg/mL)	Control	17	3.4 ± 1.51	17	2.01 ± 0.37	0.000*
	Interventional	17	3.7 ± 1.66	17	2.29 ± 0.79	0.000*
BMI* (kg/m^2^)	Control	17	23.24 ± 1.9	17	29.3 ± 3.4	0.000*
	Interventional	17	23.38 ± 1.86	17	27.74 ± 2.8	0.000*

Serum adiponectin levels (μg/mL) were significantly (p = 0.000) decreased; a value of 40.88% was discerned when assessed after a gap of three months compared to the levels prior to this period in the control group. Similarly, a significant decline (p = 0.000) of 38.11% was noticed in serum adiponectin levels in the interventional group after the VD3 intervention and three-month waiting period (Table 2).

A significant (p = 0.000) increase of 26.07% and 18.65% in BMI (kg/m^2^) value was witnessed in the control and the interventional group, respectively, after the intervention span (Table 2).

An insignificant (p = 0.511) decrease of 3.93% in serum VD3 levels was seen prior to intervention in the interventional group compared to the control. Conversely, a very significant rise of 99.41% was found in the interventional group compared to the control when assessed after the intervention (Table 3).

**Table 3:** Statistical comparison of biochemical parameters between control and interventional study groups *p-value of ≤0.05 was taken as statistically significant *BMI = body mass index

Study parameters		Control group		Interventional group		p-value
		n	Mean ± SD	n	Mean ± SD	
Vitamin D3 levels (ng/mL)	Before intervention	17	16.03 ± 4.3	17	15.4 ± 3.8	0.511
	After 3 months of intervention		17.0 ± 4.8		33.9 ± 6.2	0.000*
Adiponectin levels (μg/mL)	Before intervention	17	3.4 ± 1.51	17	3.7 ± 1.66	0.544
	After 3 months of intervention		2.01 ± 0.37		2.29 ± 0.79	0.194
BMI* (kg/m^2^)	Before intervention	17	23.24 ± 1.9	17	23.38 ± 1.86	0.83
	After 3 months of intervention		29.3 ± 3.4		27.74 ± 2.8	0.15

An insignificant (p = 0.544) upsurge of 8.82% was noted in the serum adiponectin levels of the interventional group compared to the controls before intervention. After the intervention, an insignificant (p = 0.194) decrease of 13.9% was noted in the interventional group compared to their counterpart controls (Table 3).

When the BMI of both groups was compared before the intervention, an insignificant (p = 0.83) increase of 0.60% was seen in the interventional group compared to the control group. On the other hand, an insignificant (p = 0.15) decline of 5.3% was seen in the BMI of the interventional group compared to the control after the intervention period (Table 3).

Pearson’s correlation between serum VD3 and adiponectin of the control group before intervention suggested an insignificant, weak inverse association of the two parameters (r = -0.16, p = 0.54). However, a highly significant, strong direct association was expressed between BMI and adiponectin in the controls before intervention (r = 0.83, p = 0.00004). An insignificant negative, weak association was observed between BMI and VD3 in the same study group (r = -0.07, p = 0.78). When linear correlation was performed on the control group after the three-month waiting period, an insignificant negligible inverse relation was found between VD3 and adiponectin levels (r = -0.20, p = 0.43). While in the same group, at the same time, an insignificant, very weak positive relation was explored between BMI and adiponectin levels (r = 0.013, p = 0.96). For the same group, an insignificant, very weak positive association was expressed between BMI and VD 3 levels (r = 0.075, p = 0.78) (Table 4).

**Table 4:** Pearson correlation between biochemical parameters in each study group before and after intervention *BMI = body mass index *p-value of ≤0.05 was taken as statistically significant

Study groups		Comparative study parameters	Correlation coefficient (r)	P-value
Control	Prior to intervention	Vitamin D3: Adiponectin	-0.16	0.54
		BMI*: Adiponectin	0.83	0.00004*
		BMI*: Vitamin D3	-0.07	0.78
	Post-intervention	Vitamin D3: Adiponectin	-0.20	0.43
		BMI*: Adiponectin	0.013	0.96
		BMI*: Vitamin D3	0.075	0.78
Interventional	Prior to intervention	Vitamin D3: Adiponectin	-0.088	0.74
		BMI*: Adiponectin	0.7	0.001*
		BMI*: Vitamin D3	-0.035	0.89
	Post-intervention	Vitamin D3: Adiponectin	0.273	0.28
		BMI*: Adiponectin	0.09	0.73
		BMI*: Vitamin D3	-0.12	0.65

Comparatively, the correlational association between study parameters in the interventional group before the intervention exhibited a very weak inverse insignificant association between VD3 and adiponectin serum levels (r = -0.088, p = 0.74). The same group showed a very significant direct strong relationship between BMI and adiponectin (r = 0.7, p = 0.001). Lastly, in the same group, an insignificant negligible inverse relation existed between BMI and VD3 levels (r = -0.035, p = 0.89). Following the same pattern, after the intervention of VD3 supplements along with a waiting period of three months, the interventional group expressed all insignificant associations between the study parameters. In this group, VD3 exhibited an inverse association with adiponectin (r = -0.273, p = 0.289). The same group showed a weak but direct association of BMI with adiponectin (r = 0.09, p = 0.73), and a weak inverse relationship existed between BMI and VD3 levels (r = -0.12, p = 0.65) (Table 4).

Before the intervention, linear regression analysis for the study parameter in the control group revealed that a decrease of 1.89 units of adiponectin could be predicted with an increase of one unit of VD3. However, the association was statistically insignificant (p = 0.54). In the same group, it was noticed that with the rise of one unit of BMI, an increase of 32.5 units of adiponectin could be predicted; the association was statistically very significant (p < 0.001). For the same group, the regression plot showed that with an increase of one unit of BMI, an insignificant decline of 0.23 units of VD3 could be predicted (p = 0.78) (Table 5).

**Table 5:** Linear regression analysis of biochemical parameters in each study group before and after intervention *BMI = body mass index *p-value of ≤0.05 was taken as statistically significant

Study groups		Independent variables	Dependent variables	Standardized coefficient β	R^2^ value	Predicted Ŷ equation	P-value
Control	Prior to intervention	Vitamin D3	Adiponectin	-1.89	0.026	-1.8X+144.5	0.54
		BMI*	Adiponectin	32.5	0.69	32.49X-641.27	<0.001*
		BMI	Vitamin D3	-0.23	0.0049	-0.23X+21.72	0.789
	Post-intervention	Vitamin D3	Adiponectin	-0.52	0.042	-0.5X+75.7	0.43
		BMI	Adiponectin	0.083	0.0002	0.08X +64.41	0.961
		BMI	Vitamin D3	0.19	0.0057	0.19X+11.43	0.774
Interventional	Prior to intervention	Vitamin D	Adiponectin	-1.2	0.0078	-1.2X+142.2	0.74
		BMI	Adiponectin	32.1	0.5	32.1X-626.98	0.002*
		BMI	Vitamin D3	-0.12	0.0012	-0.12X+18.08	0.894
	Post-intervention	Vitamin D3	Adiponectin	1.16	0.074	1.16X+37.3	0.289
		BMI	Adiponectin	1.8	0.0083	1.8X+26.64	0.73
		BMI	Vitamin D3	-0.54	0.013	-0.54X+48.8	0.658

In the control group, after an intervention span of three months, samples were again analyzed for a predicted association between the study parameters. All associations were proved insignificant. Specifically, it was assessed that in this group, with a one-unit upsurge of VD3, an insignificant downregulation of 0.52 units of adiponectin was predicted (p = 0.43). For the same group, the linear regression between BMI and adiponectin suggested that with a one-unit increase in BMI, only 0.083 units of adiponectin were predicted, and the association was statistically insignificant (p = 0.961). Similarly, in that group, it was observed that with a rise of one unit of BMI, an insignificant increase of 0.19 units of VD3 could be predicted (p = 0.77) (Table 5).

The regression plot for the interventional group before intervention expressed that a decline of 1.2 units of adiponectin was predicted with a rise of one unit of VD3. However, the association was insignificant, with p = 0.74. However, in the same group, it was discerned that with an increase of one unit of BMI, a significant rise of 32.1 units of adiponectin was predicted (p = 0.002). In that group, the one-unit increase in BMI suggested that an insignificant decrease of 0.12 units of VD3 could be predicted (p = 0.894) (Table 5).

In the interventional group, after the intervention, regression statistics showed that an increase of one unit of VD3 could insignificantly increase 1.16 units of adiponectin (p = 0.28). In comparison, with a rise of one unit of BMI, an insignificant upsurge of 1.8 units of adiponectin was predicted (p = 0.73). In that group, similarly, an increase of one unit of BMI predicted (p = 0.658) an insignificant decrease of 0.54 units (Table 5).

## Discussion

VD3 has developed a progressively recognized repertoire of its non-classical actions during gestation. Low VD3 in maternal mid-gestation has a significant role in the development of GDM [18]. It has been reported by Soheilykhah et al. in 2010 that as many as 83% of the women suffering from GDM had serum VD3 levels <50 nmol/ltr [19].

Previous research also suggested that reduced blood levels of VD3 in early pregnancy have been considered an independent risk factor for the development of gestational diabetes [20,21]. Recently, a Spanish study explicitly described that VD3 deficiency has a relationship with gestational diabetes, irrespective of BMI [22].

Studies reported reduced concentrations of adiponectin in association with GDM [23,24]. In 2017, an Australian study by Mousa et al. conducted with overweight or obese pregnant women who were at high risk for GDM reported an inverse relationship between baseline VD3 and adiponectin levels. This was quite similar to the results of the current study, as without the intervention of a VD3 intramuscular injection, we found the same association between VD3 and adiponectin serum levels [25]. The same Australian study also declared the mediating effect of adiponectin in high-risk pregnancies for GDM and reduced circulatory VD3 concentrations [25].

A literature review by Maysa Alzaim also emphasized the use of favorable VD3 supplemental interventions in gravid women with a VD3 deficiency to minimize the risk of status and reduce the likelihood of developing GDM. This is the same concept that we highlight in the present study, which found an accelerating effect on adiponectin by using the intervention with VD3 supplements in GDM [26].

Quite similar to the findings of the current study, another 2020 study from Iran revealed that a single intramuscular dose of VD3 injection (300,000 IU) was very much effective in not only improving the circulatory status of VD3 in mothers with GDM after delivery but it also significantly helped to boost the circulatory levels of adiponectin [14].

In line with the results of the present study, Shao et al. (2020) also reported that the results of their research conducted on Chinese pregnant women found that BMI was negatively associated with VD3 levels; that study found a reduced level of VD3 in overweight or obese pregnant women and hence declared them at high risk for diabetes mellitus [27].

Rodrigues et al. (2022) noted that in VD3-insufficient high-risk pregnancies for GDM, there was a trend of excessive weight gain in the third trimester. This agreed with the present study’s findings, as we also found a 5.6% increased average BMI in the control group compared to the interventional group in the third trimester [28].

Thagaard et al.’s 2017 Danish study observed that lower levels of adiponectin were more pronouncedly associated with those gestational diabetics who had <35 kg/m^2^ BMI, contrary to this study; our results showed an insignificant but positive association of BMI with adiponectin, as our study population owned a BMI of <28 kg/m^2^ [29].

A Chinese study by Wang et al. in 2019 concluded that VD3 may play a role in the manifestation of GDM by controlling the development of adipocytes through VD3 receptors and peroxisome proliferator-activated receptor γ (PPARγ) pathways [30].

A study from Brazil by Benaim et al. indicated that gravid women with a deficiency of serum 25 (OH)D concentrations expressed reduced serum adiponectin concentrations throughout their gestation. The present study showed that when VD3 levels were increased through intervention, a rise in serum adiponectin levels was also seen. A positive association was noticed in that group between VD3 and adiponectin [31].

The present study did not evaluate the post-partum changes in the study serum variable, which would more effectively explore the effect of GDM on these variables. Moreover, a larger sample size can present a more confident picture regarding the association of study variables. The inclusion of all categories of BMI in the study population may lead to a comparative association between different categories of BMI (underweight to obese) and variables of VD3 or adiponectin. Our study has a strength in that it included only normal to overweight BMI categories (21-25 kg/m^2^) per WHO 2017 Asia-specific criteria.

## Conclusions

In the study population of GDM pregnant women, with an increase of serum VD3 levels, a decrease in serum adiponectin level was observed; however, after the intervention of VD3 supplementation in the same subjects, with an increase in VD3, an increase in serum adiponectin level was noted. A significant positive association was seen between BMI and adiponectin in the same subjects. However, after the intervention, BMI tends to be less likely to increase adiponectin levels. Moreover, an inverse association was exhibited between BMI and VD3 levels in all the study groups, except in the control group after the intervention span, where BMI mildly raises the VD3 levels.

## Figures and Tables

**Figure 1:**
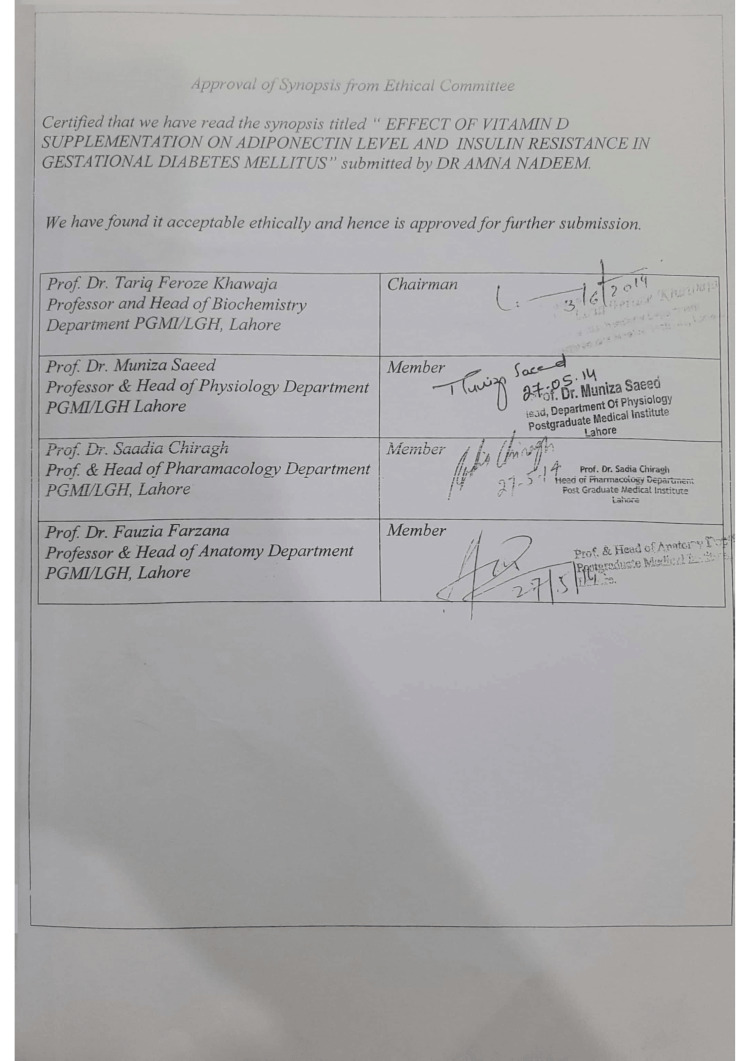
IRB approval IRB: institutional review board

## References

[1] Nguyen CL, Pham NM, Binns CW, Duong DV, Lee AH (2018). Prevalence of gestational diabetes mellitus in eastern and southeastern Asia: a systematic review and meta-analysis. J Diabetes Res.

[2] Mirghani Dirar A, Doupis J (2017). Gestational diabetes from A to Z. World J Diabetes.

[3] Kampmann U, Madsen LR, Skajaa GO, Iversen DS, Moeller N, Ovesen P (2015). Gestational diabetes: a clinical update. World J Diabetes.

[4] Inam I, Madnia E, Ahmed Ammar SS (2022). Prevalence of gestational diabetes mellitus in Pakistan: a cross sectional study. Pak J Med Health Sci.

[5] Johns EC, Denison FC, Norman JE, Reynolds RM (2018). Gestational diabetes mellitus: mechanisms, treatment, and complications. Trends Endocrinol Metab.

[6] Svensson H, Wetterling L, Bosaeus M (2016). Body fat mass and the proportion of very large adipocytes in pregnant women are associated with gestational insulin resistance. Int J Obes (Lond).

[7] Dinca M, Serban MC, Sahebkar A (2016). Does vitamin D supplementation alter plasma adipokines concentrations? A systematic review and meta-analysis of randomized controlled trials. Pharmacol Res.

[8] Mallardo M, Ferraro S, Daniele A, Nigro E (2021). GDM-complicated pregnancies: focus on adipokines. Mol Biol Rep.

[9] Williams MA, Qiu C, Muy-Rivera M, Vadachkoria S, Song T, Luthy DA (2004). Plasma adiponectin concentrations in early pregnancy and subsequent risk of gestational diabetes mellitus. J Clin Endocrinol Metab.

[10] Świrska J, Zwolak A, Dudzińska M, Matyjaszek-Matuszek B, Paszkowski T (2018). Gestational diabetes mellitus - literature review on selected cytokines and hormones of confirmed or possible role in its pathogenesis. Ginekol Pol.

[11] Retnakaran A, Retnakaran R (2012). Adiponectin in pregnancy: implications for health and disease. Curr Med Chem.

[12] Schröder-Heurich B, Springer CJ, von Versen-Höynck F (2020). Vitamin D effects on the immune system from periconception through pregnancy. Nutrients.

[13] Al-Ajlan A, Al-Musharaf S, Fouda MA (2018). Lower vitamin D levels in Saudi pregnant women are associated with higher risk of developing GDM. BMC Pregnancy Childbirth.

[14] Hosseinzadeh M, Razmpoosh E, Hosseinzadeh E (2020). The effect of a single mega dose injection of vitamin D on serum adiponectin concentration at first gestational diabetes mellitus: a randomized controlled clinical trial. Clin Nutrition Exp.

[15] Duan L, Han L, Liu Q, Zhao Y, Wang L, Wang Y (2020). Effects of vitamin D supplementation on general and central obesity: results from 20 randomized controlled trials involving apparently healthy populations. Ann Nutr Metab.

[16] Cheng Y, Chen J, Li T (2022). Maternal vitamin D status in early pregnancy and its association with gestational diabetes mellitus in Shanghai: a retrospective cohort study. BMC Pregnancy Childbirth.

[17] Kardas F, Kendirci M, Kurtoglu S (2013). Cardiometabolic risk factors related to vitamin d and adiponectin in obese children and adolescents. Int J Endocrinol.

[18] Pleskačová A, Bartáková V, Pácal L, Kuricová K, Bělobrádková J, Tomandl J, Kaňková K (2015). Vitamin D status in women with gestational diabetes mellitus during pregnancy and postpartum. Biomed Res Int.

[19] Soheilykhah S, Mojibian M, Rashidi M, Rahimi-Saghand S, Jafari F (2010). Maternal vitamin D status in gestational diabetes mellitus. Nutr Clin Pract.

[20] Lacroix M, Battista MC, Doyon M (2014). Lower vitamin D levels at first trimester are associated with higher risk of developing gestational diabetes mellitus. Acta Diabetol.

[21] Burris HH, Camargo CA Jr (2014). Vitamin D and gestational diabetes mellitus. Curr Diab Rep.

[22] Agüero-Domenech N, Jover S, Sarrión A (2021). Vitamin D deficiency and gestational diabetes mellitus in relation to body mass index. Nutrients.

[23] Noureldeen AF, Qusti SY, Al-Seeni MN, Bagais MH (2014). Maternal leptin, adiponectin, resistin, visfatin and tumor necrosis factor-alpha in normal and gestational diabetes. Indian J Clin Biochem.

[24] Thyfault JP, Hedberg EM, Anchan RM (2005). Gestational diabetes is associated with depressed adiponectin levels. J Soc Gynecol Investig.

[25] Mousa A, Abell SK, Shorakae S (2017). Relationship between vitamin D and gestational diabetes in overweight or obese pregnant women may be mediated by adiponectin. Mol Nutr Food Res.

[26] Alzaim M, Wood RJ (2013). Vitamin D and gestational diabetes mellitus. Nutrition Reviews.

[27] Shao B, Mo M, Xin X (2020). The interaction between prepregnancy BMI and gestational vitamin D deficiency on the risk of gestational diabetes mellitus subtypes with elevated fasting blood glucose. Clin Nutr.

[28] Rodrigues CZ, Cardoso MA, Maruyama JM, Neves PA, Qi L, Lourenço BH (2022). Vitamin D insufficiency, excessive weight gain, and insulin resistance during pregnancy. Nutr Metab Cardiovasc Dis.

[29] Thagaard IN, Krebs L, Holm JC, Lange T, Larsen T, Christiansen M (2017). Adiponectin and leptin as first trimester markers for gestational diabetes mellitus: a cohort study. Clin Chem Lab Med.

[30] Wang HY, She GT, Sun LZ (2019). Correlation of serum vitamin D, adipose tissue vitamin D receptor, and peroxisome proliferator-activated receptor γ in women with gestational diabetes mellitus. Chin Med J (Engl).

[31] Benaim C, Cocate PG, de Barros EG (2019). Longitudinal association of 25-hydroxyvitamin D with adipokines and markers of glucose metabolism among Brazilian pregnant women. Br J Nutr.

